# Seasonal Changes in the Nutritional Composition of *Agarophyton vermiculophyllum* (Rhodophyta, Gracilariales) from the Center of Portugal

**DOI:** 10.3390/foods10051145

**Published:** 2021-05-20

**Authors:** Clélia Afonso, Ana Patrícia Correia, Marta V. Freitas, Teresa Baptista, Marta Neves, Teresa Mouga

**Affiliations:** MARE—Marine and Environmental Sciences Centre, ESTM, Polytechnic of Leiria, Edifício CETEMARES, Av. Porto de Pesca, 2520-641 Peniche, Portugal; ana.c.correia@ipleiria.pt (A.P.C.); marta.freitas@ipleiria.pt (M.V.F.); teresa.baptista@ipleiria.pt (T.B.); marta.neves@ipleiria.pt (M.N.); mougat@ipleiria.pt (T.M.)

**Keywords:** red seaweed, protein content, fatty acid profile, amino acid profile, total dietary fiber, ash content

## Abstract

Seaweeds exhibit high nutritional value due to a balanced concentration of proteins, vitamins and minerals, a high concentration of low digestibility polysaccharides, and reduced levels of lipids, many of which are n-3 and n-6 fatty acids. The species *Agarophyton vermiculophyllum* is no exception and, as such, a comprehensive study of the chemical and nutritional profile of this red seaweed was carried out for 1 year. Seasonal variations in moisture, ash, protein and amino acids content, crude fibers, ascorbic acid, agar, lipids, and the corresponding fatty acid profile, were analyzed. We found low levels of fatty acids and a high protein content, but also noticed interesting seasonal change patterns in these compounds. The present study gives insights on the environmental conditions that can lead to changes in the nutritional composition of this species, aiming, therefore, to bring new conclusions about the manipulation of environmental conditions that allow for maximizing the nutritional value of this seaweed.

## 1. Introduction

Seaweeds are macroscopic marine algae classified into three taxa according to their accessory pigments and the nature of their polysaccharides. In this paper, we focused on the red seaweed (Rhodophyta), *Agarophyton vermiculophyllum* (Ohmi) Gurgel, J.N. Norris, formerly known as *Gracilariopsis vermiculophylla* Ohmi et Fredericq 2018. Red seaweeds exhibit phycoerythrin, phycocyanin, chlorophyll a and carotene. The main reserve polysaccharide is floridean starch, and cell wall components are cellulose, carrageenan, and agar [1,2,3]. Seaweeds are regarded as a rich source of compounds of high nutritional value due to the presence of protein, polyunsaturated fatty acids (PUFAs), polysaccharides, pigments, sterols, phenols, vitamins, and minerals, among others [1,4,5,6,7,8,9]. Due to their high nutritional value, seaweeds have been used as food for thousands of years [10,11], but also proved to have antioxidant activity [5,12,13], anti-inflammatory effect [14,15,16], immune-stimulant capacity [17,18,19], antimicrobial activity [17,20,21,22] and antitumoral effect against different types of cancer cells [16,23,24,25], among other bioactivities. Thus, due to these properties, the interest in studying them has increased greatly and seaweeds are currently being used as fodder, feed, biofuel, fertilizer, a plant growth promoter, in cosmetics, in healthcare, and in bioremediation [7,26,27,28,29]. Among red seaweeds, the *Gracilariaceae* family has achieved a relevant economic importance due to the high content of agar and its extensive use, namely by the food industry, and this includes the species *A. vermiculophyllum*, the target of our study. Regarding seaweeds’ chemical composition, in general, protein mean content ranges from 10 to 30 percent of dry matter, with red seaweeds showing the highest content with up to 47% percent in *Porphyra* and *Pyropia* sp. [1,2,30,31,32]. The most abundant amino acids in most seaweeds are aspartic and glutamic acid, as well as asparagine, serine, histidine, arginine, lysine, valine, leucine, and isoleucine [7,31,32,33]. Red seaweeds have the highest average proportion of total essential amino acids (EAA), as well as lysine, when compared to brown and green seaweeds [3]. In most seaweeds, the concentration of lipids is usually very low, ranging from 1 to 5%, with the highest concentration registered at 7.8% (dw) in the brown algae *Bellotia eriophorum* [34]. Essential fatty acids (EFA), particularly PUFAs of the n-3 and n-6 series, are abundant in seaweeds, varying from 34 to 74% of total lipid content [35]. The most common monounsaturated fatty acid (MUFA) is usually oleic acid (C18:1), reaching up to 21% of total lipids in Phaeophyceae [36]. Seaweeds also exhibit high mineral content, either macro or micronutrients, due to their ability to accumulate these from the environment [6,37,38]. Finally, they are considered as rich sources of carotenes and ascorbic acid, as their amounts may range from 20 to 170 ppm and 500 to 3000 ppm, respectively [39]. Among seaweeds, some species have achieved a relevant economic importance due to their high content in agar, an important phycocolloid used in the food and pharmaceutical industries as a thickening, stabilizing, gelling, and coating agent [40], and this includes *A. vermiculophyllum* [41].

In Portugal, the red algae *A. vermiculophyllum*, an invasive species that is found in coastal and estuarine areas, is relatively common [29,42]. This species was first reported in Portugal in Ria Formosa (36°59′ N, 7°55′ W) by Rueness in 2005 [43], but there is no information on the time it reached Lagoa de Óbidos and Figueira da Foz. *A. vermiculophyllum* seems to prefer habitats located in sheltered lagoons, bays, and estuaries, with a soft and muddy bottom, of low to moderate salinity [44], such as those found in the collection sites under study. This species is not traditionally consumed, and although it is considered edible, nutritional information is scarce. Additionally, it is well known that the chemical composition of marine seaweeds may vary considerably when they are cultivated or when collected, in different seasons, depending on temperature, light intensity, nutrients, salinity, physiological condition of the thalli, and also on the geographical distribution, lifecycle, and other ecological conditions [1,26,27,45]. Considerable regional variations also occur among the same taxon, according to the conditions the seaweed has been exposed to during growth, and specimens may vary considerably in their chemical composition.

The aim of the present work was to present a first step to a comprehensive study of the nutritional profile of *A. vermiculophyllum* collected from the center of Portugal. The study allowed us to deepen the existing knowledge about the nutritional profile of this red alga. We present results on seasonal changes in the chemical composition of *A. vermiculophyllum*, aspects that may allow us to estimate the nutritional potential of this species at different times of the year, and to provide additional information on the manipulation of the environmental conditions of cultivation to reach the full nutritional potential of this species.

## 2. Materials and Methods

### 2.1. Sampling Site, Collection, and Storage

*Agarophyton vermiculophyllum* was collected in Lagoa de Óbidos and Figueira da Foz (39°24′18.93″ N, 9°11′13.05″ W or 40°07′56.5″ N, 8°50′35.9″ W), in triplicate samples, between September 2017 and August 2018. The collection sites are characterized as transitional areas with brackish water, where *A. vermiculophyllum* is exposed to tidal cycles, over muddy substrate of the intertidal zone. Sampling took place monthly, in the intertidal zone during low tide, and transportation of the thalli to the laboratory took place in dark cooler boxes. All specimens were thoroughly washed in seawater, and epiphytes removed. Healthy portions were selected, and immediately used, freeze-dried, or dried at 25 °C in a ventilated chamber.

### 2.2. Seasonal Climatic Conditions

The season’s climatological information, which includes precipitation, radiation, and temperature data, from September 2017 to August 2018, were obtained from Climate Portal [46].

### 2.3. Estimation of Proximate Composition

#### 2.3.1. Determination of Moisture and Ash Content

Moisture and ash content were determined according to the official AOAC standard method [47]. Briefly, moisture was determined using freshly collected seaweed, blot dried, weighed, and dried at 105 °C for 48 h. Moisture content was expressed as a percentage of fresh weight (fw). Total ash was determined by incineration of the dried biomass in a muffle furnace at 525 °C for 5 h, and the results were expressed as a percentage of dry weight (dw). Iron (Fe), zinc (Zn), and calcium (Ca) contents were determined by inductively coupled plasma optical emission spectrometer (ICP-OES).

#### 2.3.2. Total Dietary Fiber Determination

Total dietary fiber was determined by the Enzymatic-Gravimetric Method, according to the AOAC Official Method 985.29 [47]. Biomass was oven dried before use and results were expressed in g 100g^−1^ dry weight.

#### 2.3.3. Total Protein Content

Nitrogen content was determined by the Kjeldahl method [47] and the total protein content was estimated using a conversion factor of 4.59, specific for red seaweeds [32]. Briefly, dried seaweed samples (0.5 g) were digested with 25 mL of 97% sulfuric acid and two catalyst tablets (Panreac, Barcelona, Spain) for 30 min at 200 °C followed by 90 min at 400 °C in a digestor apparatus (Foss Digestor 2006). Samples were cooled to room temperature, diluted with 80 mL of water, and distilled under alkaline conditions (Foss, Kjeltec 2100). The distillate was collected in 4% boric acid solution and the ammonia was quantified by titration with 0.1 M hydrogen chloride using a mix of methyl red and bromocresol green as an indicator. The protein content was calculated according to the following Equation (1).

(1) $$\mathrm{Total}\;\mathrm{protein}\;(\%)=\frac{\left(\mathrm{Vs}-\mathrm{Vb}\right)n\times 1.4\times 4.59}{m},$$

where Vs corresponds to the volume of HCl (mL) spent in the titration of the sample, Vb corresponds to the volume of HCl (mL) spent in the titration of the blank solution (prepared as a sample but without seaweed biomass), *n* corresponds to the concentration of the HCl solution used in the titration (mol L^−1^), and *m* corresponds to the initial mass (g) of the dried seaweed sample.

#### 2.3.4. Total Lipid Content

The lipid content was determined according to the Folch method [48] with modifications. A known biomass (approximately 1 g) of dried algae was mixed with 0.8 mL of water and 10 mL Folch reagent (CHCl_3_:MeOH, 2:1). The mixture was vortexed for 5 min, with 1.2 mL of 0.8% NaCl added and homogenized for 2 min. The mixture was centrifuged at 4630× *g* for 10 min at 4 °C. The lower phase was collected, filtered through a sodium sulphate anhydrous column, and collected in a weighed evaporator flask. The remaining mixture was extracted again with CHCl_3_ (5 mL), centrifuged, and the chloroform phase collected in the evaporator flask. Finally, the solvent was evaporated at 40 °C under lower pressure, until constant value. The flask containing the lipidic residue was weighed and the total lipid content was expressed in percentage and calculated according to the Equation (2).

(2) $$\mathrm{Total}\;\mathrm{Fat}\;\mathrm{Content}\;(\%)=\left(\frac{Fw-Iw}{Sw}\right)\times 100,$$

where, *Iw* is the mass (g) of the evaporator flask (g), *Fw* is the mass of flask and the lipid residue (g), and *Sw* is the mass of the dried seaweed sample (g).

#### 2.3.5. Total Carbohydrates

The total carbohydrate content was determined following the method of Dubois et al. [49]. Dried seaweed samples (100 mg) were hydrolyzed with 5 mL of hydrochloric acid 2.5 mol L^−1^, in a heating block at 100 °C for 3 h and allowed to cool to room temperature. The solution was neutralized with sodium carbonate and centrifuged at 1575× *g* for 8 min. One mL of phenol (5%) and 5 mL 96% sulfuric acid were added to 1 mL of sample solution, and the reactions were left to stand for 10 min, after which they were shaken and placed in a water bath at 25–30 °C for 20 min. Absorbance was measured at 490 nm and the concentration of carbohydrates was calculated by interpolation of a glucose calibration curve (Abs 490 nm versus glucose concentration (mg mL^−1^)). The total carbohydrate content was expressed in percentage of dry weight, according to the Equation (3).

(3) $$\mathrm{Total}\;\mathrm{Carbohydrates}\;(\%)=\left(\frac{\left(\frac{y-b}{a}\right)\times 100}{ms}\right)\times 100,$$

where *y* is the absorbance at 490 nm, b is the y-intercept value, a is the slope, m is the mass of the dried seaweed sample (mg) and V is the total volume of hydrolysate (mL).

#### 2.3.6. Agar Extraction and Quantification

The biomass was dried, weighed, powdered, and stored at −20 °C until use. Samples were hydrated in distilled water, immersed in bi-distilled water (150 mL.g^-1^) and kept in a water bath at 80 °C for 4 h. The solution was filtered in gauze and glass fiber filters (VWR, 47 mm, 1.2 μm), and the filtrate was subjected to several freeze and thaw cycles, until precipitation of the colloid, followed by a full precipitation in ethanol 96%, for 24 h. The colloid fraction was then dried at 60 °C in a drying chamber. The agar amount was measured as percentage of DW.

#### 2.3.7. Determination of Ascorbic Acid Content

Ascorbic acid concentrations were determined by the titrimetric method [47]. Briefly, 1 g of freeze-dried seaweed was added to a precipitating solution (3% metaphosphoric acid, stabilized by EDTA) and centrifuged at 10,000× *g* for 15 min. The supernatant was again centrifuged in the same conditions and 5 mL of precipitating solution was added to a standard solution of ascorbic acid (1 mg mL^−1^), followed by titration with indophenol color indicator. The concentration of vitamin C was calculated, as percentage of DW, according to the Equation (4).

(4) $$\frac{\left[mg\;of\;ascorbic\;acid\right]}{\left[g\;of\;DW\right]}=C\times V\times \frac{DF}{WT}\;,$$

where, *C* is the mg of ascorbic acid/mL of indicator, *V* is the volume of the indophenol indicator (mL) spent in the titration, *DF* is the dilution factor and *WT* is the sample dry weight in grams.

#### 2.3.8. Amino Acid Profile

Samples were previously digested through a microwave digestion system (Milestone Ethos 1) with hydrochloric acid (6 N) and phenol (0.5%) at 160 °C. The samples were neutralized with sodium hydroxide (6 N) and the final volume was set to 10mL with deionized water. Derivatization with 6-aminoquinolyl-*N*-hydroxysuccinimidyl carbamate was performed at 55 °C for 10 min. The amino acid profile was determined by an ultra-performance liquid chromatography UPLC (Waters) system equipped with a photodiode array detector (PDA). Chromatographic determination was made according to the equipment manufacturer’s instructions in an Acquity UPLC system, using eluents (Acc-Tag ultra-eluent A and B) and a BEH C18 column (2.1 mm × 100 mm, 1.7 µm) from Waters^®^ Corporation Company. A flow rate of 0.7 mL/min was used, at a constant temperature of 55 °C, with gradient conditions (0–0.54′, 99.9% A/0.1% B; 5.74′, 90.9% A/9.1% B; 7.74′, 78.8% A/21.2% B; 8.04′, 40.4% A/59.6% B; 8.70–10′, 99.9% A/0.1% B). Sample detection wavelength was at 260 nm and the amino acids were identified by comparing the retention times obtained with pure standards. The quantification was performed with a calibration curve from stock solutions containing different concentrations of each amino acid.

#### 2.3.9. Fatty Acid Analysis

The analysis of fatty acid was performed by adapting the method of Lepage and Roy [50]. To obtain the fatty acid methyl esters (FAMEs), 5 mg of crude fat were dissolved in 2.5 mL acetyl chloride: anhydrous methanol (1:20, *v*/*v*) and vortex-stirred until complete dissolution. The solution was heated at 80 °C, and 0.5 mL ultrapure water and 1 mL n-heptane were added afterwards, followed by centrifugation at 1500× *g* for 5 min. The organic upper phase was filtered and collected into GC vials. Total fatty acid profiles were acquainted by gas chromatography quantification of methyl esters (FAMEs) in a Finnigan Ultra Trace gas chromatograph equipped with a Thermo TR-FAME capillary column (60 m × 0.25 mm ID, 0.25 µm film thickness), an autosampler AS 3000 from Thermo Electron Corporation, and a flame ionization detector (FID). The temperatures of the detector and injector (operating in spitless mode) were 280 °C and 250 °C, respectively. The oven temperature gradient was 100 °C (1 min), with an increase (10 °C/min) to 160 °C (stay 10 min) and a second increase (4 °C/min) to 235 °C (during 10 min). Helium was used as carrier gas with a flow rate of 1.2 mL/min. The FID detector was supplied with synthetic air and hydrogen at flow rates of 350 and 35 mL/min, respectively. Fatty acid methyl esters were identified by the comparison of its retention times with those of the components of the Supelco 37 standard mixture (LRAB8645, Sigma-Aldrich, Bellefonte, PA, USA) analyzed on the same conditions. Each fatty acid was expressed as a percentage in respect to total peak area (TFA).

#### 2.3.10. Statistical Analysis

All the measurements were performed in triplicate, except for fiber and amino acids analysis which were *n* = 1. The data are expressed as mean ± standard deviation. Calculations were performed with SPSS Statistics 27 (IBM Corporation, Armonk, NY, USA). All the statistical analyses considered significant were at a level of 5% (*p*-value < 0.05). To test normality and variance homogeneity, the Shapiro-Wilk’s test and Levene’s F-test were used, respectively. Analysis of variance (ANOVA) and t-tests were carried out and all requirements inherent to the analysis (data normality and homogeneity of variances) were validated. If F values showed significance, a comparison was made, using post-hoc Tukey HSD tests. If the data did not fulfil these assumptions, the nonparametric Kruskal-Wallis and Mann-Whitney tests were used. Statistical analyses were performed using IBM SPSS statistical software, version 21.0 (IBM Corporation, Armonk, NY, USA).

## 3. Results

### 3.1. Seasonal Climatic Conditions

**Figure 1:**
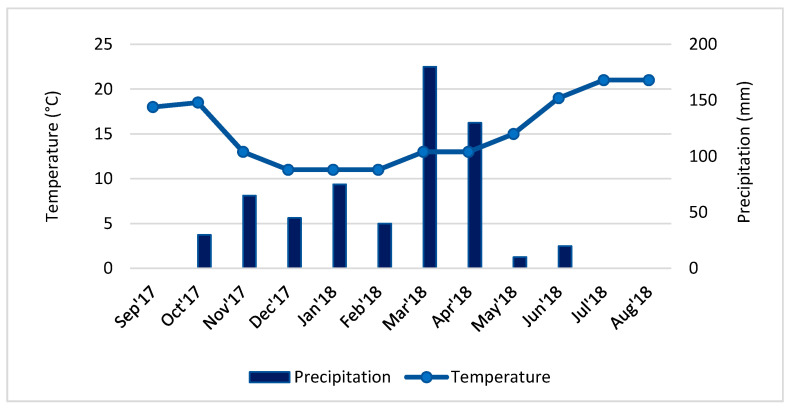
Average values of temperature and precipitation in the west center of Portugal between September 2017 and August 2018 [37].

The central west region of Portugal, where the seaweed was collected, showed precipitation and temperature in the time course under study, as seen in Figure 1. During the summer months precipitation was almost null, and temperature was around 20 °C, revealing a dry and hot climate. The 30-years average temperature and precipitation for this region in summer was 19.8 °C and 14.7 mm, respectively. In March 2018 and April 2018, during the springtime, the precipitation reached values of 180 and 130 mm, respectively, and an average air temperature of 13 °C, conditions that mark a difference between the seasons. In average terms, the water temperature varied between 14 °C in the winter months and 19 °C in summertime. The 30-years average temperature and precipitation, for this region and these months, was 11.8 °C and 90.5 mm, respectively [46]. The salinity of the water suffered little fluctuation throughout the year, revealing values around 38–39 psu. The months under study were generally drier and slightly hotter than the 30-year average, although March 2018 and April 2018 were unusually wet. Regarding incident solar radiation, the average of the last 30 years indicated maximum values for the month of July (with 264 Wm^−2^) and minimums of 57 Wm^−2^ in December. The maximum daylength was 14 h 52 m on the summer solstice, the minimum being 9 h 27 m on the winter solstice, with an average global horizontal irradiance of 1600 kWh m^−2^. 

### 3.2. Proximate Composition of Agarophyton Vermiculophyllum

The analysis of *A. vermiculophyllum* biomass, carried out in different times of the year and in two collecting sites, regarded the levels of moisture, ash, total protein, total fat, carbohydrates, total dietary fiber, agar, and ascorbic acid, as shown in Table 1.

In general, seaweeds present high moisture content and, with no exception, we found average moisture values of 75.1 ± 2.2% fw. These values were higher in October 2017 and July 2018, and lower in December 2017. Results from ANOVA and post-hoc Tukey test analysis showed that the different collection sites did not significantly change the total lipid content [F(1,28) = 1.193; *p* > 0.05], but when we consider the different months under study, there was a statistically significant differences between groups [F(9, 20) = 12.238; *p* < 0.05]. The same letters in this parameter indicate that there were no statistically significant differences in the Tukey HSD test (*p* > 0.05). 

Our results also showed that the *A. vermiculophyllum* populations under study had high ash content, reaching up to 24.5 ± 1.3% dw in September 2017, in samples collected from Figueira da Foz, with a slight but non-significant change throughout the year in both locations, indicating the natural richness of this seaweed in minerals. Iron (Fe), calcium (Ca), and zinc (Zn) contents in *A. vermiculophyllum*, averaged 96.5, 114.63, and 1.63 mg 100g^−1^ dw, respectively. Calcium reached a maximum in October 2017, but zinc and iron were higher in January 2018. Total carbohydrates were abundant, as occurs in most red seaweeds, with average values of 45.1 ± 5.9% dw, varying from 37.6 ± 2.1% in March 2018 to 48.9 ± 2.6% in October 2017, but no significant differences were found. Moreover, total dietary fiber content in *A. vermiculophyllum* averaged 28.17 ± 1.82% dw, with little change in the eight-month period under study (Table 1).

The protein content (% dw) in *A. vermiculophyllum* showed a mean value of 17.0 ± 4.5%, ranging from 21.5 ± 2.1% in September 2017 to 6.2 ± 0.3% in August 2018, showing notorious variations along the seasons, particularly in this last month (Table 1). The Mann-Whitney and Kruskal-Wallis tests showed, respectively, that there were statistically significant differences among the two locations (*U* = 61.50, *p* = 0.048) and among the different months under study (X2(9) = 25.752; *p* = 0.002) (Table 1).

As for total lipids, the results showed generally reduced levels (average of 1.7 g 100 g^−1^ dw) within the expected range for seaweeds, with a minimum of 1.2 g 100 g^−1^ dw in January 2018 and August 2018, and a maximum of 2.0 g 100 g^−1^ dw in December 2017 and April 2018 (Table 1). Results from ANOVA and post-hoc Tukey test analysis showed that the different collection sites did not influence total lipids content [F(1,27) = 0.071; *p* > 0.05], but when we consider the different months under study, there was a statistically significant difference between groups [F(9, 19) = 13.623; *p* < 0.05].

Our results also showed that in *A. vermiculophyllum*, the ascorbic acid levels were generally low (average of 0.18 mg g^−1^ dw), with a maximum of 0.28 ± 0.01 mg g^−1^ DW, in March 2018 (Table 1), showing significant differences during the analyzed time course. The Mann-Whitney and Kruskal-Wallis tests showed, respectively, that there were no statistically significant differences among the two locations (*U* = 62.50, *p* = 0.08), but there were significant differences between the months under study (X2(9) = 19.060; *p* = 0.025). The same letter in this parameter indicates that there were no statistically significant differences (*p* > 0.05) among different months.

Regarding agar, we obtained a 21.5% maximum concentration in September 2017 and a minimum of 8.8% in December 2017, with clear seasonal variation, showing statistically significant differences (*p* < 0.05). Results from ANOVA and post-hoc Tukey test analysis showed that the different collection sites did not significantly change the agar content [F(1,28) = 0.011; *p* = 0.916], but when we consider the different months under study, there was a significant difference [F(9, 20) = 3.482; *p* = 0.01].

### 3.3. Fatty Acids Analysis

The fatty acid (FA) profile in *A. vermiculophyllum*, collected in different months, is shown in Table 2. Palmitic acid (C16:0) was, in most months, the most abundant fatty acid, reaching levels up to 62.95 ± 5.69% of total FA in October 2017. In March 2018 and August 2018, arachidonic acid, a n-6 polyunsaturated fatty acid, reached a maximum (52.88 ± 3.83% and 42.14 ± 15.51%, respectively), replacing palmitic acid as the most abundant fatty acid. Besides arachidonic acid, other important EFAs were the n-6 linoleic acid (C18:2) (LA), the n-3 alpha-linolenic acid (ALA), eicosapentaenoic acid (C20:5) (EPA) and docosahexaenoic acid (C22:6) (DHA) [51]. The most common monounsaturated (MUFA) fatty acid was oleic acid (C18:1), varying from 1.33 ± 0.13 to 7.67 ± 2.84% (September 2017). Proportions of saturated (SFA), polyunsaturated (PUFAs) or monounsaturated (MUFAs) fatty acids, were set within the common values for red seaweeds, with mean values of 50.35 ± 10.10% for SFA, 11.11 ± 2.35% for MUFAs and 39.95 ± 11.19% for PUFAs. Total SFAs ranged from 33.24% in March 2018 to 64.02% in October 2017, being the most abundant group. Inversely, total PUFAs ranged from 25.82% in October 2017 to 57.57% in March 2018. Total MUFAs ranged from 7.53% in August 2017 to 14.12% in March 2018.

### 3.4. Amino Acid Profile

In *A. vermiculophyllum*, the most abundant EAA was leucine with 0.271 ± 0.034 g 100 g^−1^ fw, followed by threonine with 0.178 ± 0.023 g 100 g^−1^ fw, phenylalanine with 0.169 ± 0.022, lysine with 0.155 ± 0.023, valine with 0.154 ± 0.019, isoleucine with 0.126 ± 0.017, methionine with 0.085 ± 0.014 and, finally, histidine with only 0.053 ± 0.006 g 100 g^−1^ fw (Table 3). As for the other amino acids, glutamine was the most abundant with 0.635 ± 0.122 g 100 g^−1^ fw, followed by asparagine with 0.465 ± 0.06 g 100 g^−1^ fw. The total essential amino acids level was rather low, accounting for 1.19 g 100 g^−1^ fw.

## 4. Discussion

The nutritional composition of seaweeds change according to geographic location, species, and environmental conditions of growth, among other factors [55]. Current and future studies, which aim to intensify the gathering of information on the variability of nutritional composition, are essential to identify which seasons or harvest places are most interesting for a given species and thus direct its use in the food industry. The metabolic activity is altered by temperature, pH, and nutrient availability according to different climatic conditions, generating changes in the chemical composition of *A. vermiculophyllum* [56].

In this species, moisture content showed slight, but significant differences among the months in study. Dry matter values were in line with or slightly higher than the values found in related species by other authors (13–25%) [57,58]. As samples were collected at the intertidal zone, they were occasionally exposed to desiccation due to tidal cycles, but extreme changes were not expected [59]. The ash content also fit well in the wide range of values reported in red seaweeds (5.8–46.2% dw) [9]. Ashes reached a maximum of 24.5 ± 1.3%, indicating the relative richness in minerals when compared to *Gracilaria corticata* (8.10 ± 0.49%), *Hydropuntia edulis* (formerly *Gracilaria edulis*, 7.36 ± 0.39–8.7 ± 0.34%) or other *Gracilaria* spp. (9.25 ± 0.42–22.89 ± 1.27%) [49,50,51]. The values found for *Crassiphycus birdiae* (formerly *Gracilaria birdiae*, 9.25–22.5%), *Crassiphycus caudatus* (formerly *Gracilaria caudata*, 23%), *Gracilaria domingensis* (23.8%), and other *Gracilaria* sp. (29%), as well as *Agarophyton chilensis* (24.0 ± 0.813% dw) [33,60,61], are like the ash content found in our samples. Slight oscillation was registered for ash content throughout the year, but with no statistical significance, thus, seasonal changes did not seem to affect this parameter.

Iron (Fe), calcium (Ca) and zinc (Zn) contents were high and comparable with those found in the literature, although these vary considerably, namely for *Gracilaria* sp., *G. gracilis, Hydropuntia edulis* (formerly *Gracilaria edulis*) and *Gracilariopsis longissima* (formerly *Gracilaria verrucosa*), (Fe varying from 21.34 to 211.0 mg 100g^−1^ DW, Ca from 200.4 to 948.45 mg 100g^−1^ DW, Zn from 1.7 to 7.85 mg 100g^−1^ DW, respectively) [60,62,63]. *A. vermiculophyllum* stands out for its high iron levels and could therefore be a possible source of this mineral [64]. However, the bioavailability of iron must be evaluated, ensuring that the necessary levels of intake are acceptable, considering the levels of iodine or other minerals, that can limit its consumption as a safe source of iron. As to Ca and Zn, the levels found in 100g dw of *A. vermiculophyllum* were lower than daily requirements for humans, respectively, of 1000 mg and 11 mg for a male adult, and it is impracticable to ingest that amount of seaweed, but further analysis must be performed [65].

Usually, seaweeds are rich in dietary fiber, although fiber varies considerably among different species: in red seaweeds it varies between 5.7 to 64.7%, in green seaweeds from 29 to 66%, and in brown seaweeds from 10 to 64% in a dry weight basis [9,63,66,67,68]. High proportions of dietary fiber are generally regarded as beneficial for the promotion and maintenance of human gut health [6,69]. *A. vermiculophyllum* shows an intermediate percentage of total dietary fiber, with a mean value higher than the one found in *A. chilensis* (21.7 ± 2.4% dw) by Morales and co-workers [61], but like some *Gracilaria* species (24.7–30.5% dw) [70]. These values, though, vary considerably in gracilariods, presenting higher values in *Crassiphycus birdiae* (formerly *Gracilaria birdiae*, 52 to 61% dw), *Crassiphycus caudatus* (formerly *Gracilaria caudata*, 44.5% dw), *G. domingensis* (41.5% dw) [67] and *Hydropuntia edulis* (formerly *Gracilaria edulis*, 8.9 to 63% dw) [60,71]. As to *Grateloupia turuturu*, dietary fiber can reach values of approximately 60% [70]. These results show, thus, that the content and quality of red seaweed fiber can change according to taxa and to environmental conditions [72].

Carbohydrate content was in line with *G. gracilis* results, (from 5.38 to 88% dw), but lower than the results obtained in *A. chilensis* samples collected during summertime (62.3 ± 1.1% dw) [58,61,69,73]. Martín et al. [69] found a trend for higher production of carbohydrates during summer and spring due to higher bulk photosynthesis during this period, although we could not find statistically significant differences throughout the different months.

Seaweeds show variable protein content according to the taxa, though the highest content is generally found in red seaweeds, reaching up to 47% of dw [30,68]. In general, these values compare well with high-protein foods, such as soybean that averages 43.3% dw of protein [74]. Being a red seaweed, *A. vermiculophyllum* showed high protein content, consistent with the data found by other authors [5,55,73] (Table 2). In *Agarophyton chilense* [61], the protein content in biomass collected in Chile during the summer reached 13.3 ± 0.320% dw. This value is like the ones we found in July 2018, but higher than the values we found in August 2018. This last month should be analyzed with caution as the protein content was quite low when compared to the previous month (July 2018). Similar maximum values were found in the same species [75], but also in *G. gracilis* (20.2%) [27], *Gracilaria* sp. (24.7%) [7] and *Gracilaria cervicornis* (22.96%) [71]. In *Hydropuntia verrucosa* (formerly *Gracilaria edulis*) and *Gelidiella acerosa*, the total protein was much lower, respectively, with 6.68 ± 0.94 and 0.61 ± 0.07 mg g^−1^ dw [67,76]. Yet, we must note that most authors used a conversion factor of 6.25, which was established on the assumption that some nitrogen is used by the seaweed on other compounds besides proteins. Because this conversion factor often overestimates protein values, we used the conversion factor defined specifically for red seaweeds by Lourenço et al. [32] of 4.59. Protein concentration varied considerably throughout the year, with higher concentration during winter and lower during summer, as found by other authors [1,26,30,70], with significant statistical differences between months. Growth and photosynthesis of macroalgae depends on the availability of inorganic carbon, nitrogen, and phosphorus, but also light, and micronutrients. Nutrient availability depends on biotic and abiotic factors, and in the latter case it is important to consider factors such as temperature, light, desiccation, carbon availability, salinity, and water motion [77]. The *A. vermiculophyllum* under study is found in sheltered estuarine environments, in dense beds, and on intertidal muddy subtract where it is partially anchored. Water motion is a key factor for nutrient uptake, regulating the supply of nutrients (such as nitrogen and phosphorous), and in drier and warmer months the slow flows may reduce nitrogen supply and thus limit the protein level [78]. From March 2018 until August 2018 there was a tendency towards a gradual reduction in the levels of protein present, which may result from the lower availability of nitrogenous compounds. However, we must note that other seasonal factors may also affect the protein content, namely higher water temperature, salinity, and eutrophication [59,71].

Most seaweeds contain all EAA, but tryptophan, lysine and methionine tend to be limited [79]. Moreover, in vivo digestibility of seaweed proteins is limited [30], a fact that may compromise its effective nutritional value. In our samples (Table 3), all the amino acids present in *A. vermiculophyllum* were less abundant and present in reduced quantities when compared to soybean or quinoa, namely methionine and lysin. For example, the amount that should be consumed daily by an adult to reach the recommended intake of leucine (0.039 g/kg per day) [54] would correspond to roughly 1 kg of *A. vemicullophyllum* a day of fresh weight, for an adult weighing 70 kgs, which is much more than the recommended daily dose of seaweed (5–8 g). As for other amino acids, many seaweeds exhibit high levels of acidic amino acids (aspartic acid and glutamic acid), which can reach up to 40 percent of total amino acid content, but also high levels of asparagine, glutamine, and serine [30,31,80]. The EAA and NEAA index is around 0.51, a low limit to be considered adequate as good nutritional quality regarding amino acid content [81].

As for total lipids, the results obtained from the extraction and quantification procedures were in line with those reported previously in closely related species [1,26,33,55,61,63,82,83], showing low values (from 0.2–6.2%), but generally high quality in nutritional terms. The total lipids reached values higher than the ones found in *A. chilensis* (0.4 ± 0.056% dw) [61]. Seaweeds usually contain a minimal level of lipids, and are therefore considered a low-fat food, although higher values were found by Rosemary et al. [82] in *G. corticata* (7.07 ± 0.33% dw). We found statistically significant differences among the months under study, which apparently cannot be directly attributed to environmental factors such as temperature (Figure 1), but instead may be due to life history stage, such as physiological or even biological factors [26,59]. The reduced content of total lipids meant that their profile, in some way, did not have a very significant meaning since the absolute values present for each fatty acid were necessarily reduced. Palmitic acid (C16:0) was usually the most abundant FA, particularly in summer and late autumn. In *A. chilensis*, collected in summertime, the most abundant FA is oleic acid (C18:1n-9), with 40.27 ± 0.0%, followed by palmitic acid (C16:0) with 26.48 ± 0.3% [61]. The fatty acid (FA) profile in *A. vermiculophyllum* collected in different months is shown in Table 2. Palmitic acid (C16:0) is, in most months, the most abundant fatty acid, but in March 2018 and August 2018, arachidonic acid, an n-6 polyunsaturated fatty acid, reached maximum values and replaced palmitic acid as the most abundant fatty acid. Besides arachidonic acid, other important EFAs were the n-6 such linoleic acid (C18:2) [LA], the n-3 alpha-linolenic acid [ALA], eicosapentaenoic acid (C20:5) [EPA] and docosahexaenoic acid (C22:6) [DHA] [84]. The most common monounsaturated (MUFA) fatty acid was oleic acid (C18:1). Proportions of saturated (SFA), polyunsaturated (PUFAs) or monounsaturated (MUFAs) fatty acids were set within the common values for red seaweeds, with SFA being the most abundant group. Inversely, total PUFAs were higher in March 2018, and total MUFAs in March 2018. The increase of PUFAs and concomitant decrease in SFAs has also been recognized by Francavilla et al. [26] in *G. gracilis*, although not during spring-summer, but rather during winter when cell membranes tend to increase tightness due to lower temperatures. As the Portuguese coast tends to exhibit mild winters, the lowest temperatures registered do not seem to significantly influence the PUFAs concentration. With respect to the levels of EPA and DHA in other red algae, the content is quite variable, with little change due to environmental factors [85]. This diversity may be due to phenotypically adapted individuals exposed to the specific environmental conditions of the place where they live, the life history stage of the seaweed, sampling season and stress conditions [86]. Other interesting results relate to total n-3 fatty acids and total n-6 fatty. It is pertinent to analyze the n-6 and n-3 ratio, since high ratios are associated with the reduced occurrence of cardiovascular, autoimmune and inflammatory diseases [87,88]. January 2018 and March 2018 showed a ratio above 20:1, but all the other months showed much lower, uninteresting values. Likewise, the determination of the hypocholesterolemic and hypercholesterolemic (h/H) FA ratio is crucial due to the importance of C14:0 and C16:0 SFA on the metabolism of cholesterol. A greater h/H ratio is directly proportional to a high PUFAs content, which is more beneficial for human health. We noted, again, the influence of seasonal factors in this ratio, with January 2018 and March 2018 being the most favorable months with beneficial ratios (above 1.21), but the reduced levels of FA found makes this species an unimportant source of this type of compound. Many red seaweeds also contain high levels of ascorbic acid, comparable to terrestrial vegetables such as tomatoes and lettuce, on a wet weight basis [4].

The ascorbic acid content in other red seaweed species measured between 3.34 and 28.5 mg 100g^−1^ fw [67,76,82,89,90]. The levels of ascorbic acid that we found in *A. vermiculophyllum* had a maximum in March 2018, with significant differences during the analyzed time course. Similar values were previously found in *G. gracilis* (0.24 mg g^−1^) [90] and, although reports indicate that the consumption of 100 g of fresh weight of seaweed provides more than the daily requirements of several vitamins [6,91], the ascorbic acid analysis of *A. vermiculophyllum* does not indicate it a source of this vitamin.

As to agar content, in some species of red algae it can reach up to 31% dw [2,92] but yield, quality, and biochemical characteristics differ seasonally and depend on the species, location, growth status of the thalli, physiological factors, and reproductive cycle [92,93]. Finally, the agar of highest quality was produced during the summer, followed by the spring, when the thalli were in active growth [92]. There was also an inverse relation between agar yield and water temperature, thus lower yield in winter was expected [69]. In fact, we obtained 21.5% maximum agar concentration in September 2017 and 8.8% in December 2017, higher values than those obtained in gracilariods by Said et al. (9.89 to 14.53%) [58], but lower than the yield obtained by Marinho-Soriano (30.0%) [94] and Martín et al. [69]. Factors such as physiological condition of the thalli, life-cycle stage, and other environmental conditions or even extraction procedures may have contributed to these results [71].

Globally, when these values are compared with common edible seaweeds such as kombu (*Saccharina japonica*) and nori (*Porphyra umbilicalis*), we can state that *A. vermiculophyllum* stands as a very interesting species alongside the other gracilariod species already studied [11]. It seems to be a potential source of nutrients for food and dietary purposes, having an interesting content of fibers, proteins, minerals, and carbohydrates, however it is important to assess not only the bioavailability of the compounds, but also the presence of compounds with toxic effect, namely iodine and heavy metals.

## 5. Conclusions

The results obtained in the present study showed that proteins, lipids, agar, and ascorbic acid in *A. vermiculophyllum* show seasonal variation patterns. This demonstrates that, seasonally, *A. vermiculophyllum* can be seen as a source of nutritional proteins and fibers, safeguarding the potential reduced digestibility that might compromise these potential benefits. Other aspects should also be considered, such as mineral content, particularly iron, and further studies should be carried out in future. Toxicological information must also be safeguarded, namely the presence of iodine and heavy metals. Thus, this study presents itself as a first stage of a larger study, reporting data and providing new insights on the seasonal changes of the chemical composition of *A. vermiculophyllum*. According to the results obtained, the end of winter and spring seem to be the most suitable times of the year to harvest *A. vermiculophyllum* in its natural environment since, at that time, we registered a maximization of its nutritional potential, namely higher levels of protein, fibers, and agar in the biomass.

## Figures and Tables

**Table 1:** Proximate composition, agar content and vitamin C levels in *A. vermiculophyllum*. Data are mean values ± standard deviation (*n* = 3, except for fiber and minerals where *n* = 1). FF—samples collected from Figueira da Foz, LO—samples collected from Lagoa de Óbidos. Different letters in uppercase indicate significant differences (*p* ˂ 0.05) among means of a given analytical parameter; n.a., data not available.

	**17 September**	**17 October**	**17 November**	**17 December**	**18 January**
Collecting site	FF	LO	FF	LO	FF
Moisture (% fw)	74.1 ± 0.5 ^ab^	76.0 ± 0.2 ^b^	74.1 ± 1.4 ^ab^	71.3 ± 0.7 ^a^	74.7 ± 1.4 ^b^
Ash (%)	24.5 ± 1.3	19.6 ± 2.7	19.6 ± 2.1	19.9 ± 1.5	19.6 ± 16
Calcium (mg 100 g^−1^ dw)	138.5	190.7	n.a.	157.9	129.8
Iron (mg 100 g^−1^ dw)	148.6	80.8	n.a.	107.2	221.3
Zinc (mg 100 g^−1^ dw)	2.1	2.1	n.a	2.0	2.6
Fiber (%)	25.15	28.26	n.a	29.76	26.37
Carbohydrates (%)	46.7 ± 3.2	48.9 ± 2.6	45.9 ± 5.6	45.2 ± 8.1	40.2 ± 5.3
Protein (%)	21.6 ± 2.1 ^e^	15.5 ± 0.5 ^bcd^	19.0 ± 1.2 ^cde^	18.9 ± 1.4 ^de^	20.8 ± 0.6 ^de^
Lipids (%)	1.8 ± 0.1 ^bcde^	1.6 ± 0.1 ^bc^	1.9 ± 0.1 ^cde^	2.0 ± 0.1 ^de^	1.2 ± 0.1 ^bcde^
Vitamin C (mg g^−1^ dw)	0.15 ± 0.0 ^a^	0.15 ± 0.03 ^a^	0.17 ± 0.2 ^ab^	0.14 ± 0.1 ^a^	0.25 ± 0.2 ^bc^
Agar (%)	21.5 ± 2.1 ^c^	18.3 ± 1.5 ^bc^	18.3 ± 2.9 ^bc^	8.8 ± 0.9 ^a^	9.9 ± 1.0 ^a^
	**18 March**	**18 April**	**18 May**	**18 July**	**18 August**
Collecting site	FF	LO	FF	FF	LO
Moisture (% fw)	75.2 ± 0.6 ^b^	74.2 ± 0.6 ^b^	76.9 ± 0.9 ^ab^	77.6 ± 0.5 ^b^	75.9 ± 0.4 ^b^
Ash (%)	22.5 ± 1.0	23.7 ± 1.1	23.9 ± 0.7	21.7 ± 0.5	21.6 ± 0.5
Calcium (mg 100 g^−1^ dw)	154.3	145.9	n.a.	188.92	138.1
Iron (mg 100 g^−1^ dw)	69.1	145.1	n.a.	94.7	63.9
Zinc (mg 100 g^−1^ dw)	1.9	2.4	n.a.	2.5	1.7
Fiber (%)	29.59	29.89	n.a.	26.35	32.75
Carbohydrates (%)	37.6 ± 2.1	47.0 ± 6.8	46.4 ± 2.7	45.1 ± 5.6	48.6 ± 0.4
Protein (%)	21.2 ± 0.2 ^e^	17.1 ± 2.0 ^cde^	15.2 ± 0.3 ^bc^	14.7 ± 0.1 ^b^	6.2 ± 0.3 ^a^
Lipids (%)	1.7 ± 0.0 ^bcd^	2.0 ± 0.1 ^e^	1.5 ± 0.1 ^ab^	1.8 ± 0.1 ^bcde^	1.2 ± 0.1 ^a^
Vitamin C (mg g^−1^ dw)	0.31 ± 0.04 ^c^	0.14 ± 0.01 ^a^	0.17 ± 0.01 ^ab^	0.18 ± 0.02 ^ab^	0.22 ± 0.04 ^abc^
Agar (%)	16.3 ± 1.4 ^abc^	17.7 ± 3.8 ^bc^	9.2 ± 1.7 ^a^	11.2 ± 0.2 ^ab^	13.6 ± 2.5 ^ab^

**Table 2:** Fatty acid composition in *A. vermiculophyllum* (as a percentage of total fatty acids, TFA). Data shown is the mean ± SD (*n* = 3); h–hypocholesterolemic (MUFAs + PUFAs); H—hypercholesterolemic (C14:0 + C16:0). Bold values represent fatty acids with at least 1% of the total fatty acid composition. n.d. = not detected.

**Fatty Acids (%TFA)**	**17 September**	**17 October**	**17 November**	**17 December**	**18 January**	**18 March**	**18 April**	**18 May**	**18 July**	**18 August**
14:0 [myristic acid]	n.d.	0.31 ± 0.02	0.39 ± 0.02	0.37 ± 0.25	0.34 ± 0.07	0.27 ± 0.01	0.22 ± 0.01	0.29 ± 0.04	0.42 ± 0.06	0.26 ± 0.00
15:0 [pentadecylic acid]	0.47 ± 0.15	0.70 ± 0.04	0.76 ± 0.08	0.74 ± 0.15	0.53 ± 0.17	0.33 ± 0.13	0.35 ± 0.04	0.51 ± 0.17	0.83 ± 0.61	0.61 ± 0.00
16:0 [palmitic acid]	**57.88 ± 5.14**	**62.95 ± 5.69**	**53.62 ± 4.12**	**50.43 ± 4.19**	**44.72 ± 9.06**	**32.35 ± 2.21**	**51.18 ± 5.19**	**39.35 ± 8.83**	**58.70 ± 5.08**	**39.75 ± 10.8**
17:0 [margaric acid]	0.30 ± 0.01	n.d.	0.25 ± 0.10	n.d.	0.19 ± 0.13	0.22 ± 0.11			n.d.	0.14 ± 0.02
20:0 [arachidic acid]	**1.13 ± 0.00**	n.d.			n.d.	0.06 ± 0.00			n.d.	
21:0 [heneicosylic acid]	n.d.	n.d.			n.d.	0.10 ± 0.05	0.11 ± 0.00	0.07 ± 0.03		
23:0 [tricosylic acid]	n.d.	0.06 ± 0.00			n.d.			n.d.		
**Sum SFA (%)**	**59.78**	**64.02**	**55.02**	**51.70**	**45.89**	**33.24**	**51.89**	**40.29**	**60.91**	**40.81**
14:1 [myristoleic acid]	**5.76 ± 0.77**	**6.16 ± 0.43**	**1.97 ± 0.08**	**4.07 ± 0.94**	**3.81 ± 0.51**	**2.85 ± 0.95**	**4.87 ± 0.62**	**4.61 ± 0.63**	**5.38 ± 1.80**	**4.45 ± 1.60**
15:1 [pentadecenoic acid]	n.d.	0.27 ± 0.06	0.27 ± 0.04	0.60 ± 0.18	0.38 ± 0.14	0.50 ± 0.48	0.20 ± 0.12	0.37 ± 0.12	0.61 ± 0.64	0.43 ± 0.00
16:1 n-7 [palmitoleic acid]	0.63 ± 0.15	n.d.	n.d.	0.21 ± 0.01	0.76 ± 0.25	0.43 ± 0.22	0.43 ± 0.25	0.44 ± 0.09	1.17 ± 0.27	0.47 ± 0.08
17:1 [heptadecenoic acid]	0.46 ± 0.03	0.48 ± 0.09	0.53 ± 0.16	0.52 ± 0.11	0.43 ± 0.10	0.33 ± 0.12	0.41 ± 0.04	0.45 ± 0.16	0.39 ± 0.05	0.24 ± 0.03
18:1 n-9 [oleic acid)	**7.67 ± 2.84**	**6.81 ± 0.61**	**7.40 ± 2.19**	**4.42 ± 1.02**	**4.46 ± 1.08**	**3.78 ± 0.18**	**4.82 ± 1.16**	**3.22 ± 2.42**	**3.14 ± 0.82**	**1.33 ± 0.13**
20:1 n-9 [eicosenoic acid]	n.d.	n.d.	0.08 ± 0.00	0.1 ± 0.04	0.11 ± 0.00	0.11 ± 0.04	0.05 ± 0.00	n.d.	0.55 ± 0.00	0.15 ± 0.04
24:1 n-9 [nervonic acid]	n.d.	0.4 ± 0.02	n.d.	**1.21 ± 1.21**	**1.22 ± 1.08**	0.40 ± 0.20	**1.06 ± 0.10**	0.69 ± 0.29	1.15 ± 0.54	0.46 ± 0.22
**Sum MUFAs (%)**	**14.52**	**14.12**	**10.25**	**11.13**	**11.17**	**8.40**	**11.84**	**9.78**	**12.39**	**7.53**
18:2 n-6 cis [linoleic acid, LA]	**4.53 ± 0.32**	**8.27 ± 2.51**	**1.85 ± 1.12**	**2.67 ± 1.21**	**1.32 ± 0.96**	**1.34 ± 0.37**	**2.87 ± 0.72**	**4.93 ± 2.12**	**5.95 ± 4.20**	**5.14 ± 4.75**
18:2 n-6 trans [linolelaidic acid]	0.36 ± 0.08	0.37 ± 0.04	0.30 ± 0.07	0.57 ± 0.09	0.63 ± 0.11	0.52 ± 0.00	**1.02 ± 0.03**	**1.17 ± 0.74**	**1.78 ± 0.13**	**1.25 ± 0.47**
18:3 n-6 [γ-linolenic acid]	n.d.	n.d.	n.d.	n.d.	n.d.	0.60 ± 0.00	0.56 ± 0.10	0.39 ± 0.11	0.99 ± 0.11	0.95 ± 0.15
18:3 n-3 [α-linolenic acid]	n.d.	n.d.	0.55 ± 0.36	0.47 ± 0.06	0.41 ± 0.01	0.33 ± 0.04	0.32 ± 0.00	0.80 ± 0.58	0.12 ± 0.00	0.46 ± 0.07
20:2 [eicosatrienoic acid]	**1.40 ± 0.41**	n.d.	n.d.	n.d.	n.d.	0.12 ± 0.00	0.03 ± 0.00	n.d.	n.d.	n.d.
20:3 n-3 [eicosatrienoic acid] 20:3 n-6 [eicosatrienoic acid] 20:4 n-6 [arachidonic acid, AA]	n.d.	**1.56 ± 0.56**	**1.34 ± 0.58**	**1.65 ± 0.08**	0.97 ± 0.00	0.60 ± 0.88	**2.67 ± 0.22**	**1.90 ± 0.13**	**1.45 ± 0.07**	**2.72 ± 0.39**
	n.d.	n.d.	n.d.	0.13 ± 0.03	1.69 ± 0.08	0.95 ± 0.78	n.d.	n.d.	0.16 ± 0.00	n.d.
	**21.83 ± 6.31**	**14.83 ± 5.55**	**27.16 ± 5.16**	**29.74 ± 4.81**	**37.9 ± 10.26**	**52.88 ± 3.83**	**27.88 ± 5.94**	**39.83 ± 8.99**	**15.79 ± 0.76**	**42.14 ± 15.51**
20:5 n-3 [eicosapentaenoic acid, EPA] 22:6 n-3 [Docosahexaenoic acid DHA]	n.d.	0.28 ± 0.00	n.d.	0.13 ± 0.01	0.21 ± 0.00	0.18 ± 0.02	0.11 ± 0.00	0.26 ± 0.07	n.d.	n.d.
	n.d.	0.51 ± 0.00	n.d.	**4.41 ± 0.00**	n.d.	n.d.	n.d.	n.d.	n.d.	0.36 ± 0.39
**Sum PUFAs (%)**	**28.12**	**25.82**	**31.20**	**39.77**	**43.13**	**57.52**	**35.46**	**49.28**	**26.24**	**53.02**
**Nutritional Index**										
Σ n-3		2.35	1.89	6.66	1.59	1.11	3.10	2.96	1.57	3.54
Σ n-6	26.72	23.98	29.31	33.11	41.54	56.41	32.36	46.32	24.67	49.48
ratio n-6/n-3		10.20	15.51	4.97	26.13	50.82	10.44	15.65	15.71	13.98
ratio h/H	0.74	0.63	0.77	1.00	1.21	2.02	0.92	1.49	0.65	1.51

**Table 3:** Amino acid profile of *A. vermiculophyllum* compared with raw green soybean, quinoa, and the FAO/WHO requirements in human diet, including essential amino acids (EAA). Data indicates the mean values ± SD. (*n* = 6; average of six months, September 2017, October 2017, December 2017, January 18, March 2018 and April 2018). Σ EAA is the sum of all essential amino acids, Σ NEAA is the sum of all non-essential amino acids. Tryptophan was not determined.

	*A. vermiculophyllum* (g 100 g^−1^ fw)	Quinoa [52] (g 100 g^−1^ fw)	Soybean [53] (g 100 g^−1^ fw)	FAO/WHO [54] (g/kg/day)
**Essential Amino Acids**				
Thr	0.178 ± 0.023	0.426 ± 0.034	0.516	0.015
Met ^(1)^	0.085 ± 0.014	0.257 ± 0.014	0.157	0.015^(1 + 3)^
Phe ^(2)^	0.169 ± 0.022	0.615 ± 0.074	0.586	0.025^(2 + 4)^
His	0.053 ± 0.006	0.475 ± 0.048	0.348	0.010
Lys	0.155 ± 0.023	0.481 ± 0.046	0.775	0.030
Val	0.154 ± 0.019	0.458 ± 0.046	0.576	0.026
Ile	0.126 ± 0.017	0.372 ± 0.031	0.570	0.020
Leu	0.271 ± 0.034	0.713 ± 0.039	0.926	0.039
**Σ EAA**	1.190 ± 0.159			
**Non-essential Amino Acids**				
Ser	0.211 ± 0.026	0.594 ± 0.072	0.721	-
Gly	0.207 ± 0.031	0.648 ± 0.048	0.539	-
Asx	0.465 ± 0.060	0.924 ± 0.049	1.508	-
Glx	0.635 ± 0.122	1.888 ± 0.079	2.433	-
Pro	0.168 ± 0.023	0.433 ± 0.034	0.607	-
Cys ^(3)^	0.007 ± 0.002	0.165 ± 0.052	0.118	-
Ala	0.278 ± 0.035	0.466 ± 0.035	0.582	-
Tyr ^(4)^	0.134 ± 0.018	0.718 ± 0.058	0.464	-
Arg	0.227 ± 0.040	1.101 ± 0.095	1.042	-
**Σ NEAA**	2.332 ± 0.357			

The numbering refers to the fact that in the last column (FAO/WHO values), the values of Met (1) and Cys (3), are expressed together (1 + 3) and the values of Phe (2) and Tyr (4), also appear grouped (2 + 4), as indicated in that column.

## Data Availability

The data presented in this study are available on request from the corresponding author [C.A.], upon reasonable request.

## References

[1] Bocanegra A., Bastida S., Benedí J., Ródenas S., Sánchez-Muniz F.J. (2009). Characteristics and nutritional and cardiovascular-health properties of seaweeds. J. Med. Food.

[2] Holdt S.L., Kraan S. (2011). Bioactive compounds in seaweed: Functional food applications and legislation. J. Appl. Phycol..

[3] Angell A.R., Angell S.F., de Nys R., Paul N.A. (2016). Seaweed as a protein source for mono-gastric livestock. Trends Food Sci. Technol..

[4] Wells M.L., Potin P., Craigie J.S., Raven J.A., Merchant S.S., Helliwell K.E., Smith A.G., Camire M.E., Brawley S.H. (2017). Algae as nutritional and functional food sources: Revisiting our understanding. J. Appl. Phycol..

[5] Kazir M., Abuhassira Y., Robin A., Nahor O., Luo J., Israel A., Golberg A., Livney Y.D. (2018). Extraction of proteins from two marine macroalgae, *Ulva* sp. and *Gracilaria* sp., for food application, and evaluating digestibility, amino acid composition and antioxidant properties of the protein concentrates. Food Hydrocoll..

[6] MaCartain P., Gill C.I.R., Brooks M., Campbell R., Rowland I.R. (2007). Nutritional Value of Edible Seaweeds. Nutr. Rev..

[7] Vieira E.F., Soares C., Machado S., Correia M., Ramalhosa M.J., Oliva-teles M.T., Paula Carvalho A., Domingues V.F., Antunes F., Oliveira T.A.C. (2018). Seaweeds from the Portuguese coast as a source of proteinaceous material: Total and free amino acid composition profile. Food Chem..

[8] Paiva L., Lima E., Neto A.I., Marcone M., Baptista J. (2017). Nutritional and Functional Bioactivity Value of Selected Azorean Macroalgae: *Ulva compressa*, *Ulva rigida*, *Gelidium microdon*, and *Pterocladiella capillacea*. J. Food Sci..

[9] Gamero-Vega G., Palacios-Palacios M., Quitral V. (2020). Nutritional Composition and Bioactive Compounds of Red Seaweed: A Mini-Review. J. Food Nutr. Res..

[10] Peñalver R., Lorenzo J.M., Ros G., Amarowicz R., Pateiro M., Nieto G. (2020). Seaweeds as a functional ingredient for a healthy diet. Mar. Drugs.

[11] Pereira L., Pomin V.H. (2011). A review of the nutrient composition of selected edible seaweeds. Seaweed: Ecology, Nutrient Composition and Medicinal Uses.

[12] Gomez-Zavaglia A., Prieto Lage M.A., Jimenez-Lopez C., Mejuto J.C., Simal-Gandara J. (2019). The potential of seaweeds as a source of functional ingredients of prebiotic and antioxidant value. Antioxidants.

[13] Hermund D.B., Qin Y. (2018). Antioxidant Properties of Seaweed-Derived Substances. Bioactive Seaweeds for Food Applications.

[14] Barbalace M.C., Malaguti M., Giusti L., Lucacchini A., Hrelia S., Angeloni C. (2019). Anti-inflammatory activities of marine algae in neurodegenerative diseases. Int. J. Mol. Sci..

[15] Yang E.-J., Ham Y., Lee W., Lee N., Hyun C.-G. (2013). Anti-inflammatory effects of apo-9′-fucoxanthinone from the brown alga, *Sargassum muticum*. DARU J. Pharm. Sci..

[16] De Jesus Raposo M.F., De Morais A.M.B., De Morais R.M.S.C. (2015). Marine polysaccharides from algae with potential biomedical applications. Mar. Drugs.

[17] Telles C.B.S., Mendes-Aguiar C., Fidelis G.P., Frasson A.P., Pereira W.O., Scortecci K.C., Camara R.B.G., Nobre L.T.D.B., Costa L.S., Tasca T. (2018). Immunomodulatory effects and antimicrobial activity of heterofucans from *Sargassum filipendula*. J. Appl. Phycol..

[18] Schleder D.D., Peruch L.G.B., Poli M.A., Ferreira T.H., Silva C.P., Andreatta E.R., Hayashi L., do Nascimento Vieira F. (2018). Effect of brown seaweeds on Pacific white shrimp growth performance, gut morphology, digestive enzymes activity and resistance to white spot virus. Aquaculture.

[19] Sakthivel M., Deivasigamani B., Rajasekar T., Kumaran S., Alagappan K. (2015). Immunostimulatory Effects of Polysaccharide Compound from Seaweed *Kappaphycus alvarezii* on Asian seabass (*Lates calcarifer*) and it’s Resistance against *Vibrio parahaemolyticus*. J. Mar. Biol. Oceanogr..

[20] Lopes G., Sousa C., Silva L.R., Pinto E., Andrade P.B., Bernardo J., Mouga T., Valentão P. (2012). Can phlorotannins purified extracts constitute a novel pharmacological alternative for microbial infections with associated inflammatory conditions?. PLoS ONE.

[21] Govindasamy C., Narayani S., Arulpriya M., Ruban P., Anantharaj K., Srinivasan R. (2011). In vitro antimicrobial activities of seaweed extracts against human pathogens. J. Pharm. Res..

[22] Capillo G., Savoca S., Costa R., Sanfilippo M., Rizzo C., Giudice A.L., Albergamo A., Rando R., Bartolomeo G., Spanò N. (2018). New Insights into the Culture Method and Antibacterial Potential of *Gracilaria gracilis*. Mar. Drugs.

[23] Yuan Y.V., Walsh N.A. (2006). Antioxidant and antiproliferative activities of extracts from a variety of edible seaweeds. Food Chem. Toxicol..

[24] Milledge J.J., Nielsen B.V., Bailey D. (2016). High-value products from macroalgae: The potential uses of the invasive brown seaweed, *Sargassum muticum*. Rev. Environ. Sci. Biotechnol..

[25] Rodrigues D., Alves C., Horta A., Pinteus S., Silva J., Culioli G., Thomas O.P., Pedrosa R. (2015). Antitumor and antimicrobial potential of bromoditerpenes isolated from the Red Alga, *Sphaerococcus coronopifolius*. Mar. Drugs.

[26] Francavilla M., Franchi M., Monteleone M., Caroppo C. (2013). The red seaweed *Gracilaria gracilis* as a multi products source. Mar. Drugs.

[27] Soares C., Machado S., Vieira E.F., Morais S., Teles M.T., Correia M., Carvalho A., Domingues V.F., Ramalhosa M.J., Delerue-Matos C. (2017). Seaweeds from the Portuguese coast: A potential food resource?. IOP Conference Series: Materials Science and Engineering.

[28] Leandro A., Pacheco D., Cotas J., Marques J.C., Pereira L., Gonçalves A.M.M. (2020). Seaweed’s bioactive candidate compounds to food industry and global food security. Life.

[29] Pacheco D., Araújo G.S., Cotas J., Gaspar R., Neto J.M., Pereira L. (2020). Invasive Seaweeds in the Iberian Peninsula: A Contribution for Food Supply. Mar. Drugs.

[30] Fleurence J. (1999). Seaweed proteins: Biochemical, nutritional aspects and potential uses. Trends Food Sci. Technol..

[31] Fleurence J., Ele Morançais M., Dumay J., Decottignies P., Turpin V., Munier M., Garcia-Bueno N., Jaouen P. (2012). What are the prospects for using seaweed in human nutrition and for marine animals raised through aquaculture?. Trends Food Sci. Technol..

[32] Lourenço S.O., Barbarino E., De-Paula J.C., Otávio L., Pereira S., Marquez U.M.L. (2002). Amino acid composition, protein content and calculation of nitrogen-to-protein conversion factors for 19 tropical seaweeds. Phycol. Res..

[33] Gressler V., Sumie Yokoya N., Fujii T., Colepicolo P., Filho J.M., Torres R.P., Pinto E. (2010). Analytical Methods Lipid, fatty acid, protein, amino acid and ash contents in four Brazilian red algae species. Food Chem..

[34] Schmid M., Kraft L.G.K., Van Der Loos L.M., Kraft G.T., Virtue P., Nichols P.D., Hurd C.L. (2018). Southern Australian seaweeds: A promising resource for omega-3 fatty acids. Food Chem..

[35] Di Pasquale M.G. (2009). The Essentials of Essential Fatty Acids. J. Diet. Suppl..

[36] Dawczynski C., Schubert R., Jahreis G. (2007). Amino acids, fatty acids, and dietary fibre in edible seaweed products. Food Chem..

[37] Neveux N., Bolton J.J., Bruhn A., Ras M., La Barre S., Bates S.S. (2018). The Bioremediation Potential of Seaweeds: Recycling Nitrogen, Phosphorus, and Other Waste Products. Blue Biotechnology: Production and Use of Marine Molecules.

[38] Lozano Muñoz I., Díaz N.F. (2020). Minerals in edible seaweed: Health benefits and food safety issues. Crit. Rev. Food Sci. Nutr..

[39] Rajapakse N. (2011). Nutritional and Digestive Health Benefits of Seaweed. Adv. Food Nutr. Res..

[40] Cardoso S.M., Carvalho L.G., Silva P.J., Rodrigues M.S., Pereira O., Pereira L. (2014). Bioproducts From Seaweeds: A Review With Special Focus On The Iberian Peninsula. Curr. Org. Chem..

[41] Sousa A.M.M., Sereno A.M., Hilliou L., Gonçalves M.P. (2010). Biodegradable Agar extracted from *Gracilaria vermiculophylla*: Film Properties and Application to Edible Coating. Mater. Sci. Forum.

[42] Bermejo R., MacMonagail M., Heesch S., Mendes A., Edwards M., Fenton O., Knöller K., Daly E., Morrison L. (2020). The arrival of a red invasive seaweed to a nutrient over-enriched estuary increases the spatial extent of macroalgal blooms. Mar. Environ. Res..

[43] Rueness J. (2005). Life history and molecular sequences of *Gracilaria vermiculophylla* (Gracilariales, Rhodophyta), a new introduction to European waters. Phycologia.

[44] Nejrup L.B., Pedersen M.F. (2012). The effect of temporal variability in salinity on the invasive red alga *Gracilaria vermiculophylla*. Eur. J. Phycol..

[45] Shields R.J., Lupatsch I. (2012). Algae for Aquaculture and Animal Feeds. TATuP Z. Tech. Theor. Prax..

[46] (2020). Climate Portal Data. http://portaldoclima.pt/pt/.

[47] AOAC (2016). Official Methods of Analysis of AOAC International.

[48] Folch J., Lees M., Sloane Stanley G. (1957). A Simple Method for the Isolation and Purification of Total Lipides from Animal Tissues. J. Biol. Chem..

[49] Dubois M., Gilles K.A., Hamilton J.K. (1956). Colorimetric Method for Determination of Sugars and related Substances. Anal. Chem..

[50] Lepage G., Roy C.C. (1986). Direct transesterification of all classes of lipids in one-step reactions. J. Lipid Res..

[51] Simopoulos A.P. (2002). The importance of the ratio of omega-6/omega-3 essential fatty acids. Biomed. Pharmacother..

[52] Mota C., Santos M., Mauro R., Samman N., Matos A.S., Torres D., Castanheira I. (2016). Protein content and amino acids profile of pseudocereals. Food Chem..

[53] U.S. Department of Agriculture, Agricultural Research Service (2019). Food Data Central, Nutrient Data Laboratory, Beltsville Human Nutrition Research Center, ARS, USDA. https://fdc.nal.usda.gov/fdc-app.html#/food-details/169282/nutrients.

[54] WHO, FAO, UNU (2007). Protein and Amino Acid Requirements in Human Nutrition: Report of a Joint WHO/FAO/UNU Expert Consultation.

[55] Cherry P., O’Hara C., Magee P.J., Mcsorley E.M., Allsopp P.J. (2019). Risks and benefits of consuming edible seaweeds. Nutr. Rev..

[56] Samanta P., Jang S., Shin S., Kim J.K. (2020). Effects of pH on growth and biochemical responses in *Agarophyton vermiculophyllum* under different temperature conditions. J. Appl. Phycol..

[57] Rasyid A., Ardiansyah A., Pangestuti R. (2019). Nutrient Composition of Dried Seaweed *Gracilaria gracilis*. Indones. J. Mar. Sci..

[58] Ben Said R., Mensi F., Majdoub H., Ben Said A., Ben Said B., Bouraoui A. (2018). Effects of depth and initial fragment weights of *Gracilaria gracilis* on the growth, agar yield, quality, and biochemical composition. J. Appl. Phycol..

[59] Madden M., Mitra M., Ruby D. (2012). Seasonality of selected nutritional constituents of edible Delmarva Seaweeds. J. Phycol..

[60] Tabarsa M., Rezaei M., Ramezanpour Z., Waaland J.R. (2012). Chemical compositions of the marine algae *Gracilaria salicornia* (Rhodophyta) and *Ulva lactuca* (Chlorophyta) as a potential food source. J. Sci. Food Agric..

[61] Morales C., Schwartz M., Sepúlveda M., Quitral V. (2019). Composición química y propiedades tecnológicas de alga roja, *Agarophyton chilensis* (ex *Gracilaria chilensis*). Rev. Cienc. Tecnol..

[62] Nazni P., Deepa S. (2015). Minerals and Heavy metal present in the selected red seaweeds of south coast region of Tamilnadu. Int. J. Res. Biol. Sci..

[63] Debbarma J., Rao B.M., Murthy L.N., Mathew S., Venkateshwarlu G., Ravishankar C.N. (2016). Nutritional profiling of the edible seaweeds *Gracilaria edulis*, *Ulva lactuca* and *Sargassum* sp.. Indian J. Fish..

[64] National Institute of Health Iron—Fact Sheet for Health Professionals. https://ods.od.nih.gov/factsheets/Iron-HealthProfessional/.

[65] NHS Vitamins and Minerals. https://www.nhs.uk/conditions/vitamins-and-minerals/.

[66] Torres P., Santos J.P., Chow F., dos Santos D.Y.A.C. (2019). A comprehensive review of traditional uses, bioactivity potential, and chemical diversity of the genus *Gracilaria* (Gracilariales, Rhodophyta). Algal Res..

[67] Sakthivel R., Devi K.P. (2015). Evaluation of physicochemical properties, proximate and nutritional composition of *Gracilaria edulis* collected from Palk Bay. Food Chem..

[68] Raposo M.F.D.J., De Morais A.M.M.B., De Morais R.M.S.C. (2016). Emergent sources of prebiotics: Seaweeds and microalgae. Mar. Drugs.

[69] Martín L.A., Rodríguez M.C., Matulewicz M.C., Fissore E.N., Gerschenson L.N., Leonardi P.I. (2013). Seasonal variation in agar composition and properties from *Gracilaria gracilis* (Gracilariales, Rhodophyta) of the Patagonian coast of Argentina. Phycol. Res..

[70] Denis C., Morançais M., Li M., Deniaud E., Gaudin P., Wielgosz-Collin G., Barnathan G., Jaouen P., Fleurence J. (2010). Study of the chemical composition of edible red macroalgae *Grateloupia turuturu* from Brittany (France). Food Chem..

[71] Marinho-Soriano E., Fonseca P.C., Carneiro M.A.A., Moreira W.S.C. (2006). Seasonal variation in the chemical composition of two tropical seaweeds. Bioresour. Technol..

[72] Rajauria G., Cornish L., Ometto F., Msuya F.E., Villa R. (2015). Identification and selection of algae for food, feed, and fuel applications. Seaweed Sustain. Food Non Food Appl..

[73] Rodrigues D., Freitas A.C., Pereira L., Rocha-Santos T.A.P., Vasconcelos M.W., Roriz M., Rodríguez-Alcalá L.M., Gomes A.M.P., Duarte A.C. (2015). Chemical composition of red, brown and green macroalgae from Buarcos bay in Central West Coast of Portugal. Food Chem..

[74] Gorissen S.H.M., Crombag J.J.R., Senden J.M.G., Waterval W.A.H., Bierau J., Verdijk L.B., van Loon L.J.C. (2018). Protein content and amino acid composition of commercially available plant-based protein isolates. Amino Acids.

[75] Parjikolaei B.R., Bruhn A., Eybye K.L., Larsen M.M., Rasmussen M.B., Christensen K.V., Fretté X.C. (2016). Valuable Biomolecules from Nine North Atlantic Red Macroalgae: Amino Acids, Fatty Acids, Carotenoids, Minerals and Metals. Nat. Resour..

[76] Syad A.N., Shunmugiah K.P., Kasi P.D. (2013). Seaweeds as nutritional supplements: Analysis of nutritional profile, physicochemical properties and proximate composition of *G. acerosa* and *S. wightii*. Biomed. Prev. Nutr..

[77] Roleda M.Y., Hurd C.L. (2019). Seaweed nutrient physiology: Application of concepts to aquaculture and bioremediation. Phycologia.

[78] Hurd C.L. (2017). Shaken and stirred: The fundamental role of water motion in resource acquisition and seaweed productivity. Perspect. Phycol..

[79] Misurcová L. (2011). Chemical Composition of Seaweeds. Handbook Marine Macroalgae: Biotechnology Applied Phycology.

[80] Marrion O., Schwertz A., Fleurence J., Guéant J.L., Villaume C. (2003). Improvement of the digestibility of the proteins of the red alga *Palmaria palmata* by physical processes and fermentation. Nahr. Food.

[81] Brown M.R., Jeffrey S.W. (1992). Biochemical composition of microalgae from the green algal classes Chlorophyceae and Prasinophyceae. 1. Amino acids, sugars and pigments. J. Exp. Mar. Biol. Ecol..

[82] Rosemary T., Arulkumar A., Paramasivam S., Mondragon-Portocarrero A., Miranda J., Rosemary T., Arulkumar A., Paramasivam S., Mondragon-Portocarrero A., Miranda J.M. (2019). Biochemical, Micronutrient and Physicochemical Properties of the Dried Red Seaweeds *Gracilaria edulis* and *Gracilaria corticata*. Molecules.

[83] Morais T., Inácio A., Coutinho T., Ministro M., Cotas J., Pereira L., Bahcevandziev K. (2020). Seaweed potential in the animal feed: A review. J. Mar. Sci. Eng..

[84] Candela C.G., López L.B., Kohen V.L. (2011). Importance of a balanced omega 6/omega 3 ratio for the maintenance of health. Nutritional recommendations. Nutr. Hosp..

[85] Khotimchenko S., Vaskovsky V., Przhemenetskaya V. (1991). Distribution of eicosapentaenoic and arachidonic acids in different species of Gracilaria. Phytochemistry.

[86] Hafting J.T., Craigie J.S., Stengel D.B., Loureiro R.R., Buschmann A.H., Yarish C., Edwards M.D., Critchley A.T. (2015). Prospects, and challenges for industrial production of seaweed bioactives. J. Phycol..

[87] Paiva L., Lima E., Neto A.I., Baptista J. (2018). Seasonal Variability of the Biochemical Composition and Antioxidant Properties of *Fucus spiralis* at Two Azorean Islands. Mar. Drugs.

[88] WHO Interim Summary of Conclusions and Dietary Recommendations on Total Fat and Fatty Acids. Proceedings of the Joint FAO/WHO Expert Consultation on Fats and Fatty Acids in Human Nutrition.

[89] Norziah M.H., Ching C.Y. (2000). Nutritional Composition of Edible Seaweed *Gracilaria changgi*. Food Chem..

[90] Yildiz G., Dere E., Dere Ş. (2014). Comparison of the antioxidative components of some marine macroalgae from Turkey. Pak. J. Bot..

[91] Ortiz J., Romero N., Robert P., Araya J., Lopez-Hernández J., Bozzo C., Navarrete E., Osorio A., Rios A. (2006). Dietary fiber, amino acid, fatty acid and tocopherol contents of the edible seaweeds *Ulva lactuca* and *Durvillaea antarctica*. Food Chem..

[92] Gioele C., Marilena S., Valbona A., Nunziacarla S., Andrea S., Antonio M. (2017). *Gracilaria gracilis*, Source of Agar: A Short Review. Curr. Org. Chem..

[93] Pangestuti R., Kim S.K. (2015). Seaweed proteins, peptides, and amino acids. Seaweed Sustainability: Food and Non-Food Applications.

[94] Marinho-Soriano E. (2001). Agar polysaccharides from *Gracilaria species* (Rhodophyta, Gracilariaceae). J. Biotechnol..

